# Large-Scale Transcriptome Data Analysis Identifies KIF2C as a Potential Therapeutic Target Associated With Immune Infiltration in Prostate Cancer

**DOI:** 10.3389/fimmu.2022.905259

**Published:** 2022-06-03

**Authors:** Pingxin Zhang, Hang Gao, Chunwei Ye, Ruping Yan, Lu Yu, Chengxing Xia, Delin Yang

**Affiliations:** Department of Urology, The Second Affiliated Hospital of Kunming Medical University, Kunming, China

**Keywords:** prostate cancer, KIF2C, pan-cancer, immune infiltration, prognostic biomarker, TME

## Abstract

Prostate cancer (PCa) is one of the most prevalent cancers of the urinary system. In previous research, Kinesin family member 2C (KIF2C), as an oncogene, has been demonstrated to have a key role in the incidence and progression of different cancers. However, KIF2C has not been reported in PCa. We combined data from different databases, including The Cancer Genome Atlas, the Cancer Cell Line Encyclopedia, Genotype Tissue-Expression, cBioPortal, and the Genomics of Drug Sensitivity in Cancer database, to explore the potential oncogenic role of KIF2C in PCa through a series of bioinformatics approaches, including analysis of the association between KIF2C and prognosis, clinicopathological features, gene mutations, DNA methylation, immune cell infiltration, and drug resistance. The results showed that KIF2C was significantly up-regulated in PCa. High KIF2C expression was associated with age, pathological stage, lymph node metastases, prostate-specific antigen (PSA), and Gleason score and significantly predicted an unfavorable prognosis in PCa patients. Results from Gene Set Enrichment Analysis (GSEA) suggested that KIF2C was involved in the cell cycle and immune response. KIF2C DNA methylation was reduced in PCa and was inversely linked with KIF2C expression. KIF2C was shown to have a strong relationship with the tumor microenvironment (TME), infiltrating cells, and immune checkpoint genes. Furthermore, high KIF2C expression was significantly resistant to a variety of MAPK signaling pathway-related inhibitors. Our study reveals that KIF2C may be a possible predictive biomarker for assessing prognosis in PCa patients with immune infiltration.

## Introduction

Prostate cancer (PCa) is one of the most widespread cancers of the male genitourinary system, with a predicted 1.3 million occurrences globally in 2018 (1). The second greatest cause of cancer-related mortality in the U.S. is PCa (2), with an estimated 174,650 new cases in 2019. The incidence of metastatic PCa is on the rise (3). In the United States, the incidence of metastatic PCa increased from 4% in 2003 to 8% in 2017 (4). Androgen deprivation therapy (ADT) is the recommended therapy of choice for newly diagnosed progressive PCa and recurrence following radical therapy. ADT induces tumor remission at first, but resistance develops over time, leading to recurrence and ultimate progression to castration-resistant prostate cancer (CRPC) (5). CRPC patients have a terrible prognosis, with a median survival time of 9 to 30 months (6, 7). Chemotherapy, radium 223, and medicines that target the androgen receptor axis, such as enzalutamide and abiraterone, are the most common therapies for metastatic CRPC (8, 9). However, CRPC is still incurable and is the leading cause of mortality in PCa patients.

Of the kinesin superfamily, the kinesin-13 family members are strictly MT depolymerases. The most obvious member of the kinesin family-13 is kinesin family member 2C (KIF2C), also called mitotic centromere-associated kinesin (MCAK) (10). KIF2C is distributed across the cell, but is particularly abundant around centromeres, kinetochores, and spindle poles (11, 12). KIF2C is a kinesin-like protein that acts as a microtubule-dependent molecular motor during mitosis, allowing chromosomal separation (13, 14). Furthermore, both during interphase and mitosis, KIF2C modulates microtubule dynamics in the cell (15, 16). With respect to KIF2C, changes in expression have been linked to a poor prognosis for cancer recovery in several studies. In a number of malignancies, KIF2C has been recognized as a potential oncogene. According to Mo et al. (17), KIF2C enhanced hepatocellular cancer through the Ras/MAPK and PI3K/Akt signaling pathways. According to Yang et al. (18), KIF2C may accelerate the growth of cervical cancer by blocking the stimulation of the p53 signaling pathway. According to Wei et al. (19), Wnt/catenin signaling directly upregulates KIF2C expression and KIF2C overexpression causes mTORC1 pathway activation. According to An et al. (20), KIF2C increased cancer growth and was linked to tumor immune cell infiltration in endometrial cancer. According to Ha et al. (21), KIF2C promotes tumor cell motility and invasion. Furthermore, Zhu et al. suggested that KIF2C is a new player in the DNA damage response (22). However, the function of KIF2C in PCa has not been reported, which means it needs to be explored.

The goal of this research is to figure out what KIF2C’s role and mechanism in PCa could be *via* integrating multiple bioinformatics approaches. We discovered that when comparing PCa tissues to nontumor tissues, KIF2C expression was considerably higher in PCa tissues. In addition, KIF2C expression was increased with the age, stage, grade, PSA, Gleason score, lymph node metastases, and distant metastases of the PCa. High KIF2C expression was correlated with a poor prognosis for patients with PCa. Using Gene Set Enrichment Analysis (GSEA), we investigated the biological function and pathways of KIF2C. Moreover, we evaluated the possible connections between KIF2C expression and DNA methylation, gene mutations, TME, immune infiltration levels, various immune-related genes, and drug resistance in PCa. The results indicated that KIF2C expression was significantly negatively correlated with promoter methylation and positively correlated with immune scores and estimate scores. Furthermore, there was a link between KIF2C expression and the levels of B cells, T cells, macrophages, NK cells, and dendritic cell infiltration in PCa. Finally, our findings highlight the critical function of KIF2C in tumorigenesis and suggest that KIF2C may be involved in the modulation of immune responses in PCa.

## Methods

### Data Collection and Analysis of Differential Expression

Pan-cancer sequencing data and linked clinical data were downloaded from The Cancer Genome Atlas (TCGA) by the online tool UCSC Xena (https://xena.ucsc.edu/). The GTEx database (https://commonfund.nih.gov/GTEx) was used to get gene expression data for normal tissues. The CCLE database (https://portals.broadinstitute.org/ccle/) was used to gather gene expression data in tumor cell lines. Using the data downloaded above, we evaluated KIF2C expression in 31 normal tissues, 30 tumor cell lines, and 33 tumor tissues and compared KIF2C expression levels in 33 cancer samples and corresponding paracancer samples. For these tumor types, expression data was Log2 transformed, and a two-group t-test was performed. *P* < 0.05 indicated that KIF2C was differentially expressed between tumor tissues and normal tissues. All data was statistically analyzed using R software (version 4.0.2; https://www.R-project.org/), and box plots were created using the R package “ggpubr.”

### Specimen Collection

This study obtained tumor samples and related paracrine tissues from 14 pairs of PCa patients at Kunming Medical University’s Second Affiliated Hospital. None of the patients had had any chemotherapy, radiation, or endocrine treatment prior to surgery. These samples were extracted from the patients, frozen in liquid nitrogen, and kept indefinitely. All samples were donated only for the purpose of study. The Rational Committee of Kunming Medical University’s Second Affiliated Hospital gave their approval to this study.

### RNA Extraction and Quantitative Real-Time PCR (qRT-PCR)

TRIzol reagent (Sigma-Aldrich, USA) was used to extract total RNA from tissues, and quantitative real-time PCR (qRT-PCR) was performed as directed by the manufacturer. The concentration and purity of RNA were measured using a nanophotometer (IMPLEN, Germany). The iScriptTM cDNA Synthesis Kit (Promega, USA) was used to synthesize cDNA. In the qRT-PCR test, the Eastep qPCR Master Mix (Promega, USA) and the CFX96 Real-Time PCR Detection System (Bio-Rad, USA) were utilized. The primers were created by BioSune (Shanghai, China). The optimal RNA expression levels were determined using the 2^-△△Ct^ method. qRT-PCR was used to determine the expression levels of GAPDH (forward, 5′‐TGCACCACCAACTGCTTAGC‐3′, reverse, 5′‐GGCATGGACTGTGGTCATGAG‐3′) and KIF2C (forward, 5′‐CTGTTTCCCGGTCTCGCTATC‐3′, reverse,5′‐AGAAGCTGTAAGAGTTCTGGGT‐3′). All of the trials were repeated at least three times.

### Analysis of the Relationships Between KIF2C and Prognosis

Overall survival data was downloaded from the TCGA website. The connection between KIF2C expression and overall survival (OS), disease-specific survival (DSS), disease-free interval (DFI), and progression-free interval (PFI) in each cancer type was investigated using Cox regression analysis. The Kaplan–Meier techniques and the log-rank test were used to construct a survival analysis for PCa patients. To create and analyze survival curves, the R packages “survival” and “survminer” were used. To illustrate the Cox regression model, the R packages “survival” and “forestplot” were used. Significant was defined as a *P* value of less than 0.05.

### Correlation of KIF2C Expression With Gene Mutation and DNA Methylation

Gene mutation data was downloaded from TCGA by an online tool, UCSC Xena. cBioPortal (www.cbioportal.org) was used to examine the genomic alteration types and frequency of KIF2C in PCa. The gene mutations in the KIF2C high-expression group and low-expression group in PCa were drawn using the R package “maftools.” Using forest plots, we showed the differences in gene mutations between the KIF2C high expression group and the KIF2C low expression group. HM450 methylation data from cBioPortal was also utilised. For PCa, we looked at the relationship between KIF2C expression and gene promoter methylation.

### KIF2C-Related Gene Enrichment Analysis in PCa

The biology and functional relevance of KIF2C in PCa was assessed using Gene Set Enrichment Analysis (GSEA). The GSEA website (https://www.gsea-msigdb.org/gsea/downloads.jsp) was used to obtain the Reactome gene sets. GSEA was conducted using the R package Cluster Profiler.

### The Correlation of the KIF2C Expression With TME and Tumor Cell Infiltration

TME metagenes construction and gene signature scoring were carried out in accordance with previous literature (23). The stromal score, immune scores, estimated scores, and tumor purity for each tumor sample in PCa were evaluated using ESTIMATE (Estimation of Stromal and Immune Cells in Malignant Tumor Tissues Using Expression Data). The R software packages “estimate” and “limma” were used to assess the relationship between KIF2C expression level and these four indicators based on the degree of immune infiltration. The data for the infiltration score and the 24 immune cell types in the TCGA PRAD were estimated and obtained from the ImmuCellAI database (http://bioinfo.life.hust.edu.cn/ImmuCellAI#!/). CIBERSORT was used to calculate relative scores for 24 immune cells in PCa. R-packages “ggplot2”, “ggpubr,” and “ggExtra” were used to estimate the relationship between KIF2C expression levels and each immune cell infiltration level in PCa. Significance was considered for *P* values below 0.05. A co-expression study of KIF2C and immune-related genes, including genes encoding major histocompatibility complex (MHC), immune activation, immunosuppressive, chemokine, and chemokine receptor proteins, was also performed using the R-package “limma.” The “reshape2” and “RColorBreyer” packages were used to illustrate the findings.

### KIF2C and Drug Resistance

The Genomics of Drug Sensitivity in Cancer (GDSC) database (https://www.cancerrxgene.org/) collects the sensitivity and response of tumor cells to drugs and describes the response of about 200 anticancer drugs to more than 1,000 tumor cells. The GDSC database contains two datasets, GDSC1 and GDSC2. We used cell line expression profiling data for GDSC2 to analyze the relationship between KIF2C expression levels and the IC50 of 198 drugs.

### Statistical Analysis

T-tests were used to evaluate changes in KIF2C expression levels in cancer and normal tissues. All survival analyses were conducted using the Kaplan-Meier curve, the log-rank test, and the Cox proportional hazard regression model. The Spearman’s or Pearson’s test was performed to analyze the correlation between the two variables. The significance threshold for all statistical analyses was set at *P <* 0.05.

## Results

### KIF2C Expression in Different Cancers

**Figure 1:**
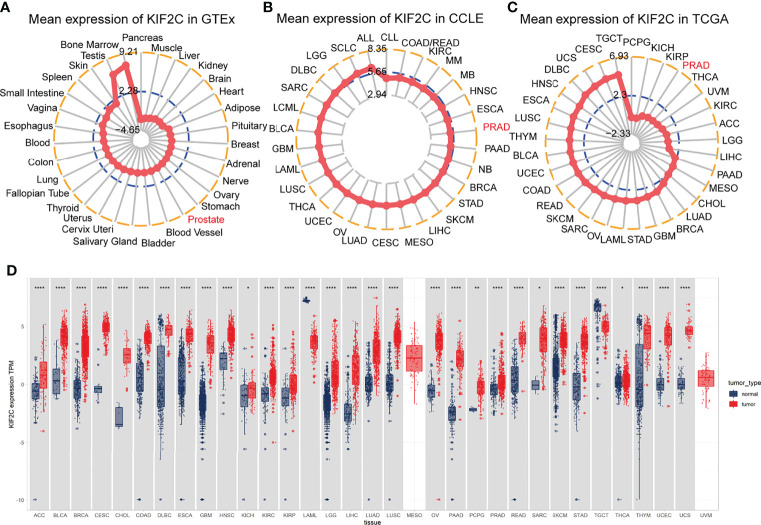
Differential expression of KIF2C. **(A)** KIF2C expression in normal tissues. **(B)** KIF2C expression in tumor cell lines. **(C)** KIF2C expression in 33 types of tumors. **(D)** Comparison of KIF2C expression between tumor and normal samples. *P < 0.05, **P < 0.01, ***P < 0.001, ****P < 0.0001.

We evaluated the physiologic expression of the KIF2C gene across human tissues from the GTEx resource (**Figure 1A**). KIF2C was highly expressed in bone marrow and testis tissues, while ubiquitously low expression was observed in most other normal tissues. We analyzed data from the CCLE to determine the relative mRNA levels of KIF2C across lineages (**Figure 1B**). According to the data from CCLE, KIF2C was highly expressed in different cancer cells. By analyzing TCGA data, we found that KIF2C expression levels were generally high in 33 types of tumor tissues (**Figure 1C**), consistent with the results of analysis of CCLE gene expression data. Next, we next compared the expression levels of KIF2C in matched normal and malignant tissue samples *via* GTEx and TCGA (**Figure 1D**). Comparisons of KIF2C expression between normal tissues and tumor samples across 33 types of cancers showed strikingly upregulated KIF2C expression among 28 types of tumor tissues. Collectively, these results demonstrate that KIF2C expression is upregulated and suggest that KIF2C may play a crucial regulatory role in various tumors’ progression.

### KIF2C Expression and Clinical Parameters of PCa Patients

**Figure 2:**
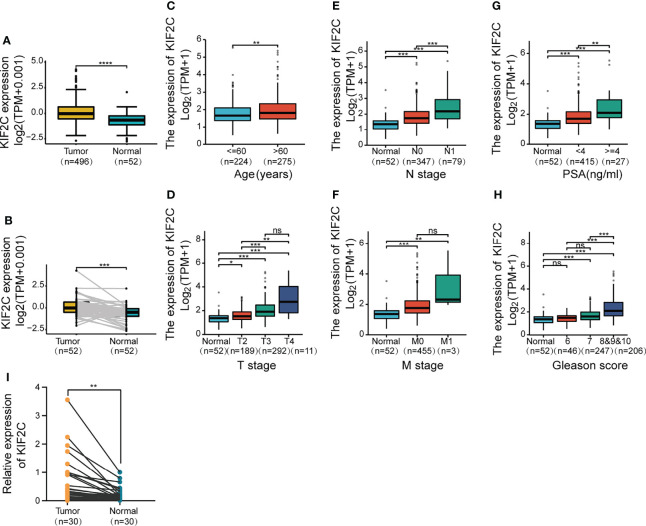
Based on the TCGA database, correlations between KIF2C expression and the key clinical indicators of PCa patients. **(A)** KIF2C expression in PCa and surrounding normal tissues. **(B)** KIF2C expression in 52 pairs of PCa tumors and surrounding normal tissues was analyzed. **(C–H)** Box plots comparing the expression of KIF2C in various groups of prostate cancer patients depending on clinical factors. **(I)** KIF2C expression in 30 paired prostate cancers and paracancerous tissues. PSA, Prostate Specific Antigen; ns, no significance; ^∗^ *p* < 0.05, ^∗∗^ *p* < 0.01, ^∗∗∗^ *p* < 0.001, and ^∗∗∗∗^ *p* < 0.0001.

Given the results of the pan-cancer analysis described above, we further analyzed the potential role of KIF2C in PCa. Using the TCGA prostate adenocarcinoma (PRAD) dataset, patients were divided into high and low expression groups based on the median value of KIF2C expression to see if there was an association between KIF2C expression and PCa clinicopathological features. In the TCGA-PRAD cohort, there were 249 patients in the KIF2C high expression group and 250 patients in the KIF2C low expression group. We found that KIF2C was associated with T-stage, N-stage, Gleason score, and PSA (**Table 1**). Next, we further analyzed the expression of KIF2C in TCGA-PRAD and compared it with normal tissues. The expression of KIF2C mRNA was observed to be higher in tumor tissues (**Figure 2A**). In addition, when 52 paired tumor samples were compared to adjacent normal samples, KIF2C expression was found to be significantly higher in PRAD (**Figure 2B**). Furthermore, we examined KIF2C expression in distinct patient groups based on clinical characteristics. KIF2C levels were shown to be higher in the PCa tissues of patients of various ages, depending on their age (**Figure 2C**). In consideration of tumor stage, KIF2C expression was shown to be significantly higher in prostate patients in stages 2, 3, and 4 (**Figure 2D**). KIF2C expression was higher among patients classified as N0 and N1 based on cancer stage (**Figure 2E**). Regarding tumor metastasis stage, KIF2C expression was higher in patients classified as M0 and M1 (**Figure 2F**). Following that, we discovered that KIF2C expression was significantly increased in PSA and Gleason score (**Figures 2G, H** **)**. In addition, we collected 30 paired PCa and paraneoplastic tissues. KIF2C was found to be significantly overexpressed in PCa tissues, according to qRT-PCR results (**Figure 2I**). Altogether, these findings indicate that KIF2C expression is enriched in PCa and might be a promising biomarker in PCa progression.

**Table 1:** Association between KIF2C mRNA expression and clinicopathologic characteristics in TCGA cohort.

Characteristic	Low expression of KIF2C	High expression of KIF2C	*p*-value
n	249	250	
Age, n (%)			0.116
<=60	121 (48.6%)	103 (41.2%)	
>60	128 (51.4%)	147 (58.8%)	
T stage, n (%)			<0.001
T2	129 (51.8%)	60 (24%)	
T3	113 (45.4%)	179 (71.6%)	
T4	3 (1.2%)	8 (3.2%)	
Unkown	4 (1.6%)	3 (1.2%)	
N stage, n (%)			<0.001
N0	176 (70.7%)	171 (68.4%)	
N1	22 (8.8%)	57 (22.8%)	
Unkown	51 (20.5%)	22 (8.8%)	
M stage, n (%)			0.249
M0	224 (90%)	231 (92.4%)	
M1	0 (0%)	3 (1.2%)	
Unkown	25 (10%)	16 (6.4%)	
Gleason score, n (%) (%) (%)			<0.001
6	38 (15.3%)	8 (3.2%)	
7	152 (61%)	95 (38%)	
8	25 (10%)	39 (15.6%)	
9	33 (13.3%)	105 (42%)	
10	1 (0.4%)	3 (1.2%)	
PSA (ng/ml), n (%)			0.011
<4	221 (88.8%)	194 (77.6%)	
>=4	7 (2.8%)	20 (8%)	
Unkown	21 (8.4)	36 (14.4%)	

### Elevated KIF2C Expression Associates With Worse Prognosis in PCa Patients

**Figure 3:**
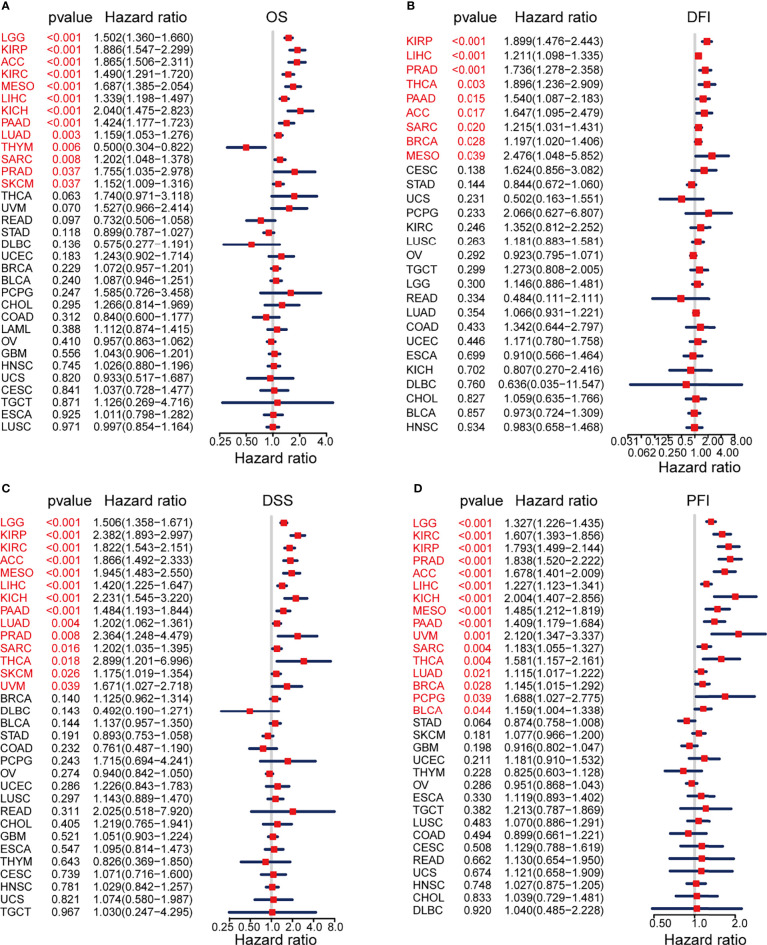
Prognostic Value of KIF2C Across Cancers. **(A)** Forest plot showing the relationship between KIF2C expression and overall survival (OS) in 33 different tumor types. **(B)** A forest plot depicting the relationship between KIF2C expression and disease-free interval (DFI) in 33 different tumor types. **(C)** A forest plot depicting the relationship between KIF2C expression and disease-specific survival (DSS) in 33 different tumor types. **(D)** Forest plot of TREM2 expression and progression-free interval (PFI) in 33 different tumor types.

**Figure 4:**
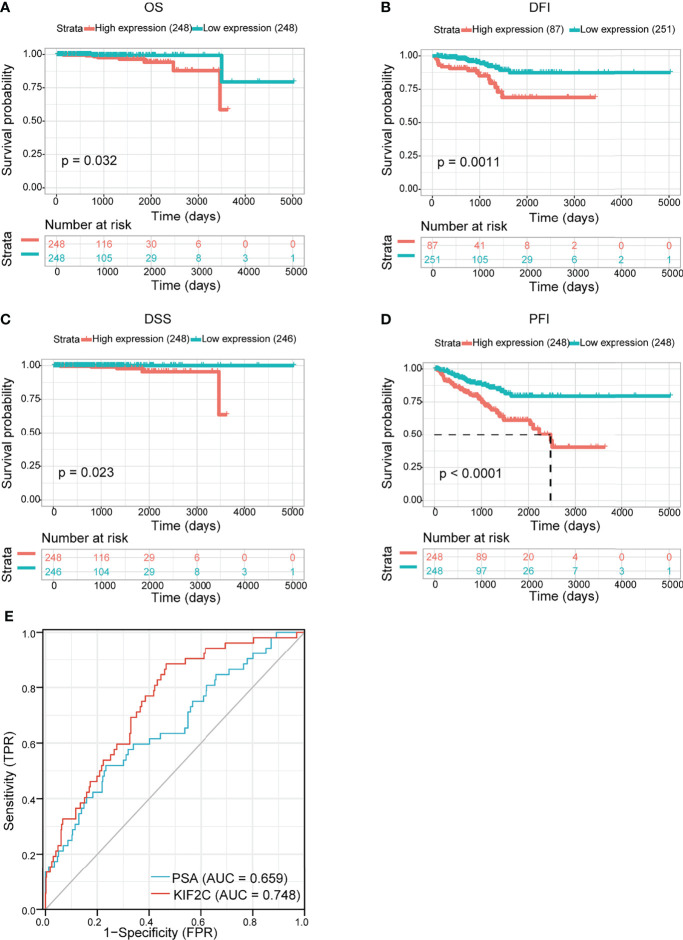
Evaluating the prognostic and diagnostic value of KIF2C in PCa. **(A–D)** The relationship between KIF2C expression and OS, DFI, DSS, and PFI was studied using the Kaplan-Meier method. **(E)** ROC curves predict KIF2C as a biomarker of PCa.

We evaluated the prognostic and diagnostic utility of the KIF2C gene since it was significantly expressed in PCa cells and tissues and was closely linked to PCa development and metastasis. In the pan-cancer dataset, we calculated the relationship between KIF2C expression and patient prognosis. Overall survival (OS), disease-free interval (DFI), disease-specific survival (DSS), and progression-free interval (PFI) are provided for each of the 33 tumor types. The Cox proportional hazards model analysis suggested that the KIF2C expression was significantly correlated with OS (*p* = 0.037), DSS (*p* = 0.008), DFI (*p* < 0.001), and PFI (*p* < 0.001) in PCa (**Figures 3A–D**). Further, KIF2C was a high-risk gene in OS (hazard ratio = 1.755), DSS (hazard ratio = 2.364), DFI (hazard ratio = 1.736) and PFI (hazard ratio = 1.838) in PCa (**Figures 3A–D**). The results from According to the Kaplan–Meier analysis, increased KIF2C expression was significantly correlated with poor OS, DSS, DFI, and PFI in PCa (**Figures 4A–D**), consistent with the results of the analysis of the Cox proportional hazards model in PCa. In addition, ROC curve analysis showed that KIF2C was an effective predictor of PCa in TCGA with an AUC of 0.748, and its diagnostic efficacy was higher than that of PSA (**Figure 4E**).

### Correlation of KIF2C Expression With DNA Methylation and Genetic Alteration

**Figure 5:**
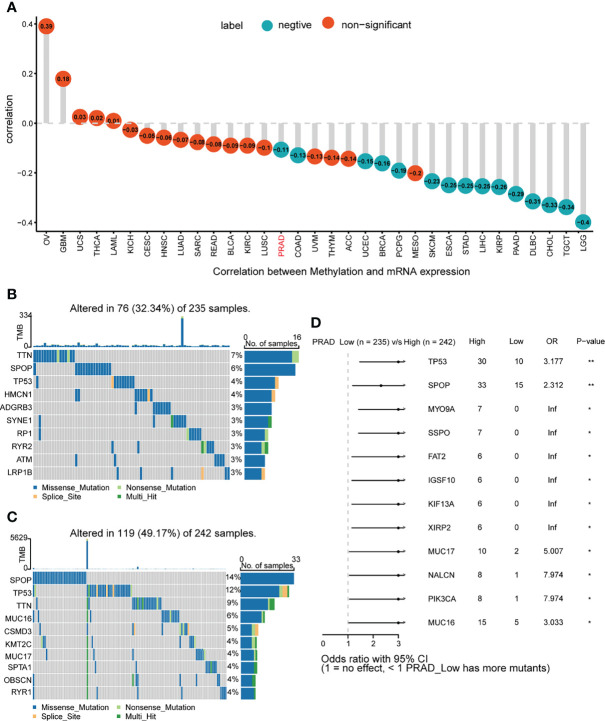
Gene promoter methylation and mutation features of KIF2C. **(A)** Correlation between KIF2C expression and gene promoter methylation in pan-cancer. **(B)** Mutated genes in PCa patients with low KIF2C expression. **(C)** Mutated genes in PCa patients with high KIF2C expression. **(D)** Comparison of mutated genes between KIF2C low expression group and KIF2C high expression group in PCa patients. ^∗^ *p* < 0.05, ^∗∗^ *p* < 0.01.

We calculated the levels of correlation between KIF2C and promoter methylation in 33 types of cancer using cBioPortal web-based data sets. Our results suggested that KIF2C expression and promoter methylation had a significant negative correlation in PRAD, COAD, UCEC, BRCA, PCPG, SKCM, ESCA, STAD, LIHC, KIRP, PAAD, DLBC, CHOL, TGCT, and LGG (**Figure 5A**). Using the cBioPortal (TCGA, Pan-Cancer Atlas) database, the pan-cancer alterations of KIF2C were investigated. These results indicated that amplification and mutation were the most common types among the different types of genetic alterations of KIF2C (**Supplement Figure 1**). In addition, using the USUC XENA database, we further analyzed the gene mutation status of KIF2C in high and low expression groups in PCa. In the KIF2C low expression group, the main mutated genes were TNN, SPOP, TP53, HMCN1, ADGRB3, SYNE1, RP1, RYR2, ATM, and LRP1B (**Figure 5B**); in the KIF2C high expression group, the main mutant genes were SPOP, TP53, TTN, MUC16, CSMD3, KMT2C, MUC17, SPTA1, OBSCA, and RYR1 (**Figure 5C**). The results of the forest plot showed that the TP53, SPOP, MYO9A, SSPO, FAT2, IGSF10, KIF13A, XIRP2, MUC17, NALCN, PIK3CA, and MUC16 genes were significantly different mutant genes in the KIF2C high and low expression groups in PCa (**Figure 5D**).

### Functional Enrichment Analysis of KIF2C in PCa

**Figure 6:**
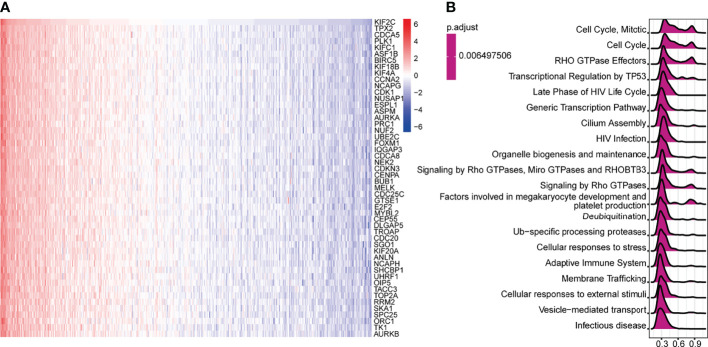
Gene Set Enrichment Analysis (GSEA) for KIF2C in PCa. **(A)** Heat maps depicting the top 50 genes in PCa that are positively linked with KIF2C. **(B)** Top 20 pathways enriched in the GSEA-Reactome analysis in PCa.

We queried the database of TCGA to identify co-expressed genes with KIF2C. Heatmaps were created for the top-50 genes that were strongly positively linked with KIF2C (**Figure 6A**). Next, a total of 300 genes positively related to KIF2C were performed using classical GSEA for PCa to explore the KIF2C-related canonical signaling pathway and biological functions. Among the GSEA results for reactome terms, the top 20 terms are shown in **Figure 6B**. Pathway analysis showed that KIF2C mainly participated in the cell cycle and several immune functional gene sets, including megakaryocyte development, platelet production, adaptive immune system, cellular responses to stress and HIV infection. In summary, these results strongly suggest an important role for KIF2C in the cell cycle and immune response in PCa.

### Correlation Analysis Between KIF2C Expression and TME in PCa

**Figure 7:**
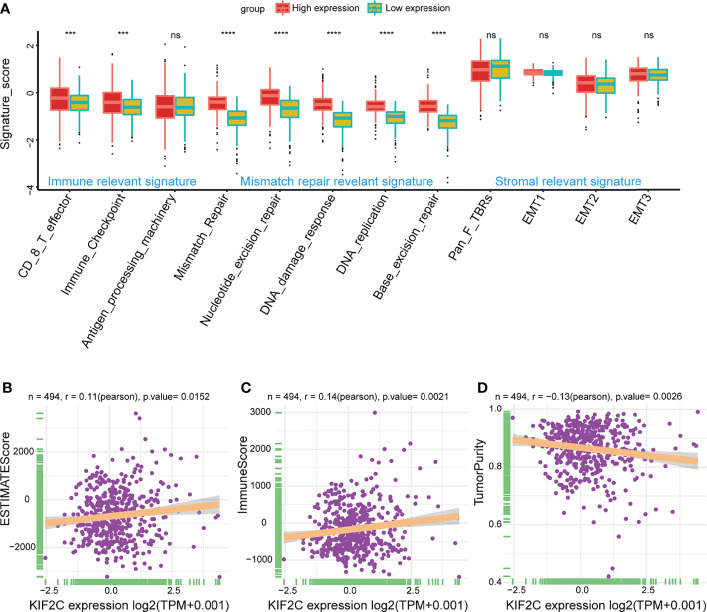
Correlation of KIF2C expression with TME. **(A)** High expression group and low expression group were distinguished by different signatures (immune relevant signature, mismatch relevant signature, and stromal relevant signature). The dispersed dots within each group reflect the mean value of signature genes. The median value is shown by the thick line. The 25th and 75th percentiles are at the bottom and top of the boxes, respectively (interquartile range). 1.5 times the interquartile range is covered by the whiskers. Asterisks indicate the range of P values above each boxplot. **(B)** Correlation between KIF2C and estimate scores in PCa. **(C)** Correlation between KIF2C and immune scores in PCa. **(D)** Correlation between KIF2C and tumor purity in PCa. ***P < 0.001, ****P < 0.0001; ns, no significance.

The tumor immune microenvironment is gradually being recognized as playing a vital role in the occurrence and development of PCa. To better characterize the function of TME signature genes, we tested known signatures in the PRAD dataset. The analysis confirmed that high KIF2C expression was significantly associated with the immune relevant signature and mismatch repair relevant signature, while the stromal relevant signature was not significantly associated (**Figure 7A**); the results for other malignancies are given in a heat map (**Supplementary Figure 2A)**. We also used the ESTIMATE algorithm to produce immune cell scores, estimate scores, and tumor purity in 33 different types of cancer, and looked at the relationship between KIF2C expression levels and these three scores. Strikingly, in PCa, our findings showed that KIF2C expression was favorably connected with immunological and estimation scores but negatively correlated with tumor purity in PCa (**Figures 7B–D**); the outcomes for other malignancies are given in a heat map (**Supplementary Figure 2B**). These findings suggest that KIF2C plays a critical role in TME.

### Relationship of the Expression of KIF2C and Tumor Immune Cell Infiltration in PCa

**Figure 8:**
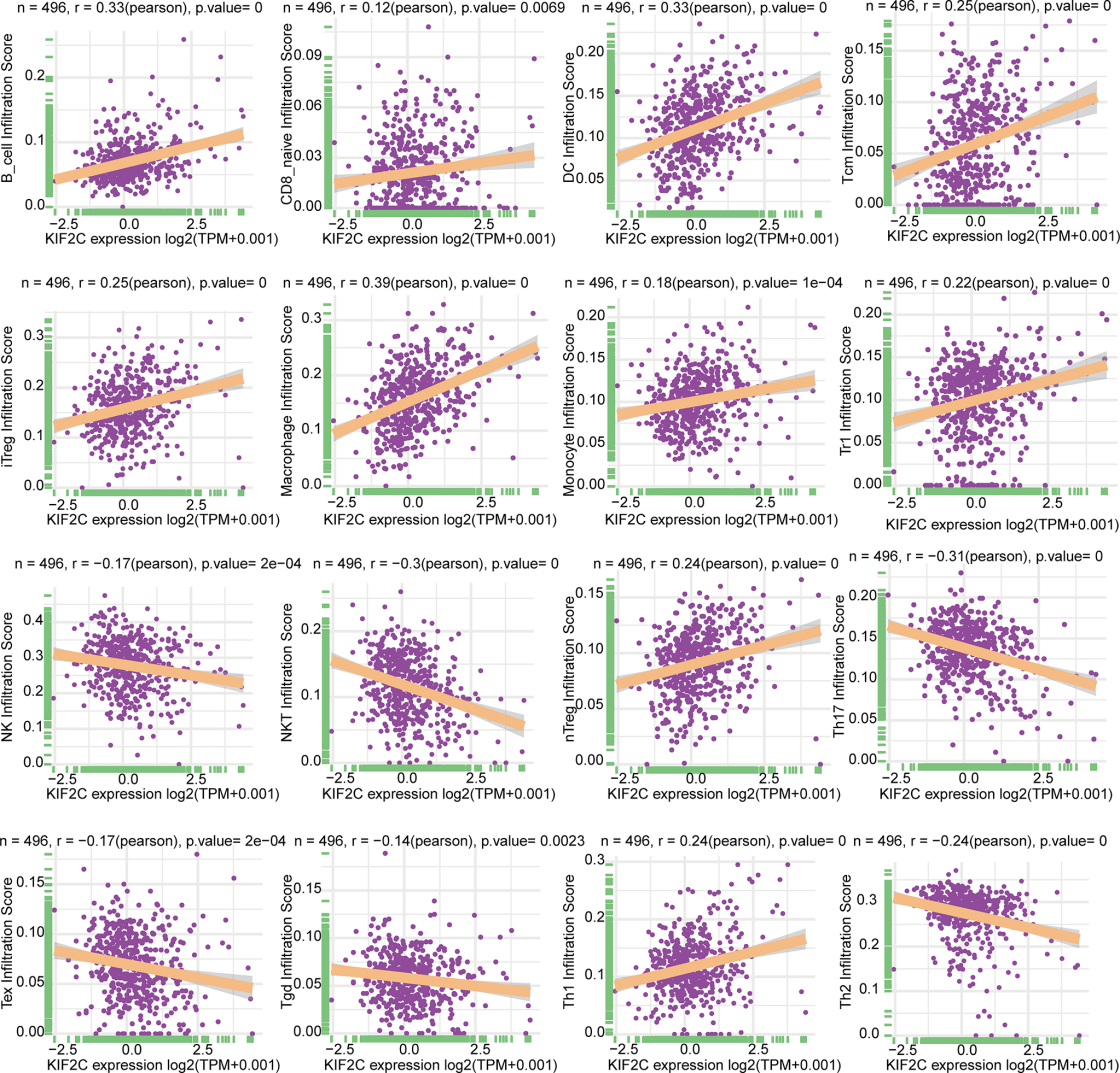
Relationship between KIF2C expression and tumor infiltration of different immune cells in PCa.

**Figure 9:**
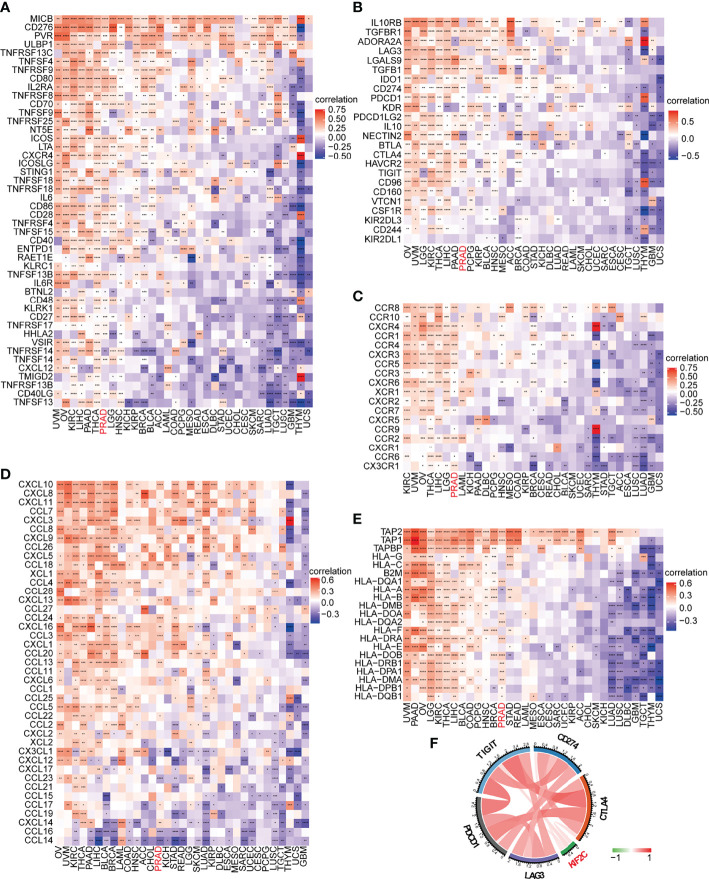
KIF2C expression was analyzed in connection to immune-related genes and immune checkpoint genes. **(A)** In pan-cancer, there is a link between KIF2C and immune activation genes. **(B)** In pan-cancer, there is a link between KIF2C and immune suppressive genes. **(C)** In pan-cancer, there is a link between KIF2C and chemokine receptors. **(D)** In pan-cancer, there is a link between KIF2C and chemokines. **(E)** In pan-cancer, the KIF2C and MHC genes are linked. **(F)** In PCa, KIF2C expression is linked to immune checkpoint members. *P < 0.05, **P < 0.01, ***P < 0.001, ****P < 0.0001.

On the basis of the ImmuCellAI database, we then did a pan-cancer analysis of the connection between KIF2C expression and 24 types of invading immune cells in 32 cancer types using the CIBERSORT approach. In PCa, our results indicated that KIF2C expression levels had a significant and negative correlation with levels of infiltrating NK cells, NKT cells, Tex, Tgd, Th2 and Th17; however, it was positively correlated with levels of infiltrating B cells, CD8+ nave cells, dendritic cells, iTreg cells, macrophages, monocytes, Tr1, nTreg, Tcm, and Th1 (**Figure 8**). The relationships between KIF2C expression and infiltrating immune cells in other tumors are shown in the heat map (**Supplementary Figure 3**). We also used gene co-expression analyses to look at the correlations between KIF2C expression and immune-related genes to see if KIF2C and other immune modulators play a synergistic role in PCa. Investigated were MHC, immunological activation, immunosuppression, chemokine, and chemokine receptor genes. According to the heatmap data (**Figures 9A–E**), almost all immune-related genes were co-expressed with KIF2C, and the majority were positively associated with KIF2C in PCa. Additionally, to further investigate the synergistic role of KIF2C in PCa-induced immune responses, we studied the correlation of KIF2C with other immune checkpoint members. We found that KIF2C expression correlated strongly with LAG3, PDCD1, TIGIT, CD274 and CTLA4 (**Figure 9F**).

### KIF2C and Drug Resistance

**Figure 10:**
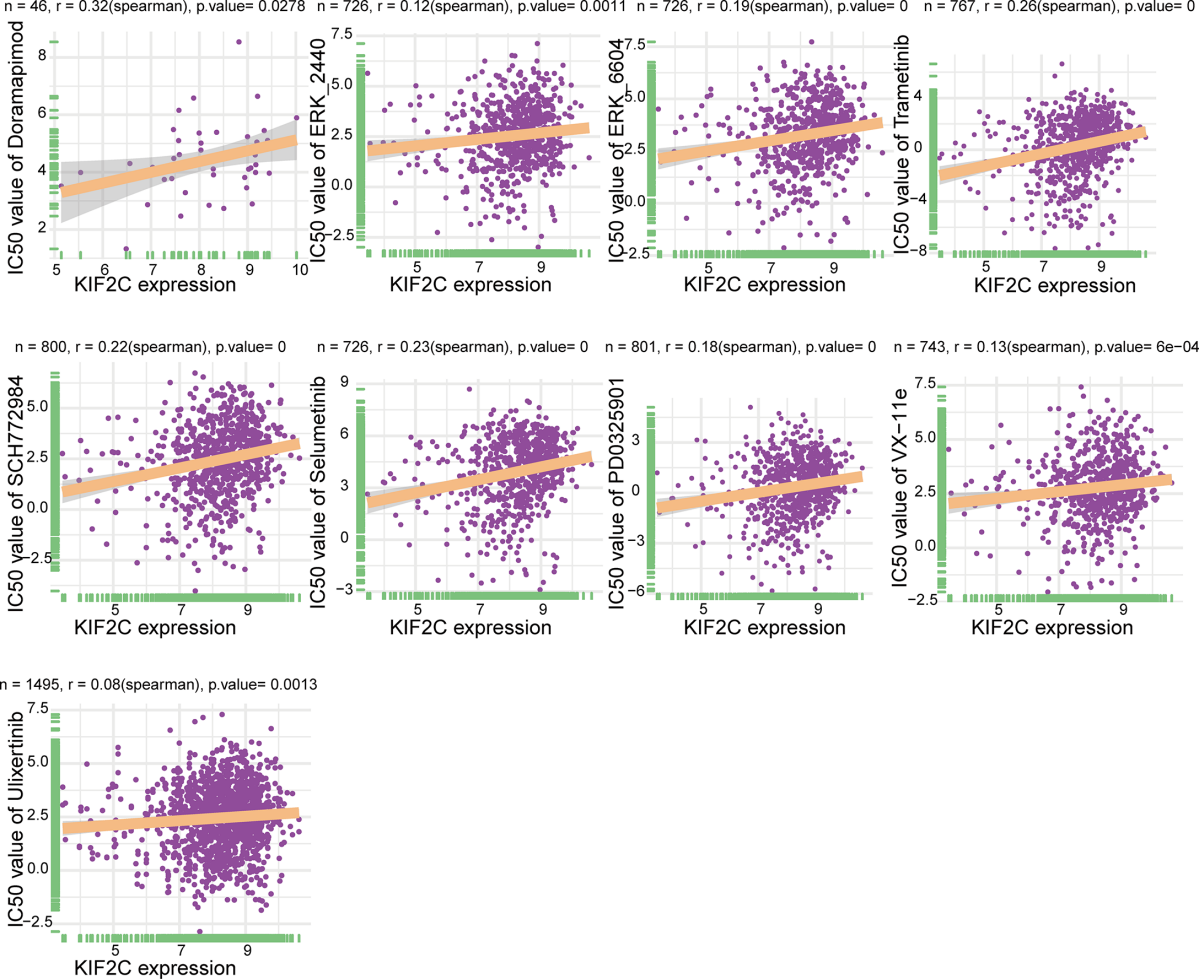
An illustration of the relationship between KIF2C expression and the IC50 of drugs.

An important clinical issue in cancer treatment is drug resistance. Here, we used GDSC2 datasets to analyze the relationship between KIF2C expression levels and IC50 of 198 drugs to explore drug resistance due to high KIF2C expression in PCa. Notably, our results suggested that high KIF2C expression was involved in resistance to many drugs, including doramapimod, ERK2440, ERK6604, trametinib, SCH772984, selumetinib, PD0325901, ulixertinib, and VX-11e (**Figure 10**). Interestingly, these drugs were all MAPK signaling pathway inhibitors. In summary, our results provide new perspectives and new horizons for PCa treatment.

## Discussion

PCa is the fifth highest cause of cancer death in the male population and is among the most prevalent cancers of the urinary system (24, 25). There is currently a lack of effective biomarkers for PCa diagnosis and prognosis. KIF2C, a member of the Kinesin 13 family, is mainly involved in processes such as spindle assembly, chromosome aggregation and segregation, and centromere-microtubule attachment, and is essential for mitosis. Previous studies have shown that KIF2C as an oncogene plays an important role in the occurrence and development of various tumors. However, KIF2C has not been reported in PCa. In our research, we comprehensively investigated the expression, prognosis, functional enrichment of KIF2C in PCa, and the association between KIF2C expression and DNA methylation, clinical characteristics, immune response, and drug resistance.

Previous research has revealed that KIF2C is substantially expressed in liver cancer (17, 19, 26, 27), breast cancer (28), endometrial carcinoma (20), lung cancer (29, 30), glioma (31), bladder cancer (32), colorectal cancer (33), esophageal squamous cell carcinoma (34), cervical cancer (18), and thyroid cancer (35). The results of our pan-cancer analysis indicated that KIF2C was highly expressed in 28 tumors, including PCa, which was consistent with previous results reported in the literature. For the first time, we detected the expression of KIF2C in PCa tissue and corresponding paracancerous tissue, and the results showed that KIF2C was significantly more expressed in PCa tissue than in paracancerous tissue. The expression of KIF2C is significantly correlated with clinical and pathologic tumor features. According to Yang et al. (18), KIF2C expression was linked to the kind of cervical cancer. In hepatocellular carcinoma, Mo et al. found that KIF2C was substantially linked with pathological stage and neoplasm histological grade (17). According to Gan et al., elevated KIF2C expression in NSCLC tissues was associated with a higher T stage, poor differentiation status, and lymph node metastases (29). In our study, we also showed that high expression of KIF2C was associated with an increase in tumor stage, Gleason score, PSA score, lymph node metastasis, and distant metastasis in PCa. In addition, our pan-cancer analysis of the prognostic indicated that KIF2C was significantly associated with poor prognosis in different tumor types and was a high-risk factor affecting tumor prognosis. In PCa, patients with high KIF2C expression had significantly worse OS, DSS, DFI, and PFI. The above results reveal that KIF2C plays an oncogenic role in PCa.

The occurrence and progression of cancer are linked to gene mutations (36). We further explored the characteristics of KIF2C gene mutations. Yang et al. found that the most common KIF2C mutation was a missense mutation, and that the mutation was linked to the survival rate of cervical squamous cell carcinoma (18). Mike et al. discovered that boosting WT MCAK/Kif2C protein levels over indigenous MCAK/Kif2C promoted chromosomal instability in a comparable manner (37). The results of our pan-cancer analysis indicated that KIF2C had different degrees of genetic variation in 26 cancer types, including mutations, structural variants, amplification, and deep deletion. Mutation and amplification are types of KIF2C genetic variation in PCa. It is unclear whether a genetic variant of the KIF2C gene was associated with a poor prognosis for patients with PCa. PCa progression is often accompanied by mutations in genes that add to the difficulty of treating PCa (38). Therefore, a role-shaping analysis of PCa gene mutations and misregulation will help to understand, diagnose, and better treat PCa. Our findings show that patients with high KIF2C expression are accompanied by high-frequency mutations in genes such as SPOP, TP53, and TTN. Previous studies have shown that mutations in these genes are often associated with a poor prognosis for PCa (39–41). Genetic testing for PCa patients is now evolving, allowing clinicians to develop individualized management strategies based on patient-specific genetic alterations. Knowledge of these genetic mutations can better help clinicians assess the malignancy, progression, and prognosis of PCa patients and provide treatment guidelines for PCa patients. DNA methylation is a common type of epigenetic alteration that controls gene expression without changing the DNA sequence (42). By altering chromatin structure, DNA stability, and DNA conformation, DNA methylation normally decreases gene expression (43). In recent decades, researchers have steadily identified correlations between DNA methylation and cancer. In malignant cells, hypermethylation within promoter regions frequently results in the silence or inactivation of tumor suppressor genes. The link between KIF2C DNA methylation and KIF2C expression has never been studied before. Our results indicated for the first time that KIF2C DNA methylation was significantly inversely associated with KIF2C expression in PRAD, COAD, UCEC, BRCA, PCPG, SKCM, ESCA, STAD, LIHC, KIRP, PAAD, DLBC, CHOL, TGCT, and LGG. However, the regulatory relationship between levels of KIF2C DNA methylation and KIF2C expression levels is complex and merits further investigation.

The immune system is essential in the body’s defense system, playing a critical role in cancer development and progression (44). In recent years, cancer immunotherapies have ushered in a new age in tumor treatment, with long-term clinical results (45). Androgen receptors are important for the growth and survival of PCa cells. Androgen deprivation therapy is a cornerstone of PCa treatment aimed at reducing tumor size by blocking androgen signaling. Studies have reported that ADT has a close regulatory relationship with CD4+ and CD8+ T lymphocytes, natural killer cells, macrophages, and T regulatory (Treg) cells (46–49). However, PCa tumors are weakly immunogenic, and immunotherapy for PCa still has many limitations that require further exploration. Screening antibodies in colorectal cancer have identified NY-CO-58/KIF2C as a tumor antigen (33). An et al. study found that knocking down KIF2C decreased CD8+ T cell apoptosis, which was further inhibited when paired with anti-PD1 in endometrial cancer (20). However, the relationship between KIF2C and immune response has not been explored in PCa. Our results indicate that KIF2C plays a critical role in PCa immunity. It is well-known that the TME has dramatic effects on the outcome of tumor growth (42, 50–52). Our results suggest that high KIF2C expression was mainly linked to CD8 T effectors and immune checkpoints in the TME. In addition, there were positive correlations between KIF2C expression and both immune scores and estimated scores in the TME of PCa. Immune cells infiltrating tumors have a significant impact on tumor formation and progression (53). Our results suggest that KIF2C is closely associated with a variety of immune-infiltrating cells in PCa, including T cells, NK cells, B lineage cells, dendritic cells, macrophages, monocytes, et al. We also found that KIF2C has a significant positive correlation with immune checkpoints such as LAG3, PDCD1, TIGIT, CD274, and CTLA4 in PCa. Furthermore, KIF2C was found to be co-expressed with genes encoding MHC, immunological activation, immunosuppression, chemokines, and chemokine receptor proteins in our research. In the current study, we first demonstrated that the expression of KIF2C was closely related to the immune response, providing a new perspective for immunotherapy of the prostate.

The mitogen-activated protein kinase (MAPK) pathway, including the kinases RAS, RAF, MEK, and ERK, regulates tumor cell proliferation, apoptosis, inflammation, angiogenesis, metastasis, and drug resistance, and plays an important role in cancer development (54). Several drugs that inhibit the MAPK pathway have been developed in preclinical and clinical settings and have shown promising efficacy and anticancer activity in the treatment of several types of malignancies (55). It has been reported that there is a relationship between KIF2C and the MAPK signaling pathway. According to Mo et al. (17), the downregulated oncogene KIF2C suppressed the development of hepatocellular carcinoma through the Ras/MAPK signaling pathway. Furthermore, in a transformed model, knocking down K-Ras or inhibiting ERK1/2 activation lowered KIF2C expression, suggesting that KIF2C might be a potential target for cancer medication treatment (56). Our study showed that high KIF2C expression leads to resistance to multiple MAPK signaling pathway inhibitors, such as Doramapimod, ERK2440, ERK6604, trametinib, SCH772984, selumetinib, PD0325901, ulixertinib, and VX-11e. This provides new insights into the pharmacological treatment of tumors. Although the MAPK signaling pathway is critical in the progression of PCa, inhibitors of the MAPK signaling pathway as well as the regulatory relationship between KIF2C and the MAPK signaling pathway have not been reported in PCa. Therefore, the phenomenon of high KIF2C expression leading to resistance to multiple MAPK signaling pathway inhibitors in PCa deserves further exploration.

In summary, our first PCa analyses of KIF2C indicated that this factor was closely associated with the malignancy and can be used as a risk prognostic factor in PCa. KIF2C expression has been linked to immune cell infiltration, TME, and immune checkpoint activation. Moreover, high KIF2C expression was involved in resistance to MAPK signaling pathway inhibitors. These investigations may help further shed light on the role of KIF2C in PCa progression and expansion and can provide a more accurate reference for the realization of immunotherapy in the future. However, the study had several limitations. First, the majority of the studies in this study were based on KIF2C mRNA levels. The findings would be more persuasive if they were analyzed further depending on protein levels. Second, we did not explore the prognostic value of KIF2C methylation in PCa in this study. Third, the relationship between KIF2C and immune response and the relationship between KIF2C and drug resistance needs to be further proved *in vivo* and *in vitro* experiments.

## Data Availabity Statement

The datasets presented in this study can be found in online repositories. The names of the repository/repositories and accession number(s) can be found in the article/**Supplementary Material**.

## Ethics Statement

The studies involving human participants were reviewed and approved by Kunming Medical University’s Second Affiliated Hospital. The patients/participants provided their written informed consent to participate in this study.

## Author Contributions

The study was conceived by DY. PZ and CX wrote the manuscript and conducted the research. The literature search and data collection were done by HG, CY, RY, and LY. PZ, CX, and HG all contributed to the manuscript’s drafting and data interpretation. The essay was co-authored by all writers, who all gave their approval to the final edition.

## Funding

The Natural Science Foundation of China (No. 81860453), the Yunnan Provincial Major Science and Technology Projects (No. 2018ZF009), and the Yunnan Applied Basic Research Project [No. 2018FE001(-006)] funded this research.

## Conflict of Interest

The authors declare that the research was conducted in the absence of any commercial or financial relationships that could be construed as a potential conflict of interest.

## Publisher’s Note

All claims expressed in this article are solely those of the authors and do not necessarily represent those of their affiliated organizations, or those of the publisher, the editors and the reviewers. Any product that may be evaluated in this article, or claim that may be made by its manufacturer, is not guaranteed or endorsed by the publisher.
