# Supplementary material for: Large-Scale Transcriptome Data Analysis Identifies KIF2C as a Potential Therapeutic Target Associated With Immune Infiltration in Prostate Cancer
Source: Front Immunol. 2022 Jun 3;13:905259. doi: 10.3389/fimmu.2022.905259 (PMC9203693; doi:10.3389/fimmu.2022.905259)
Supplement: Supplementary file 1 [file DataSheet_1.docx]

Supplementary Material


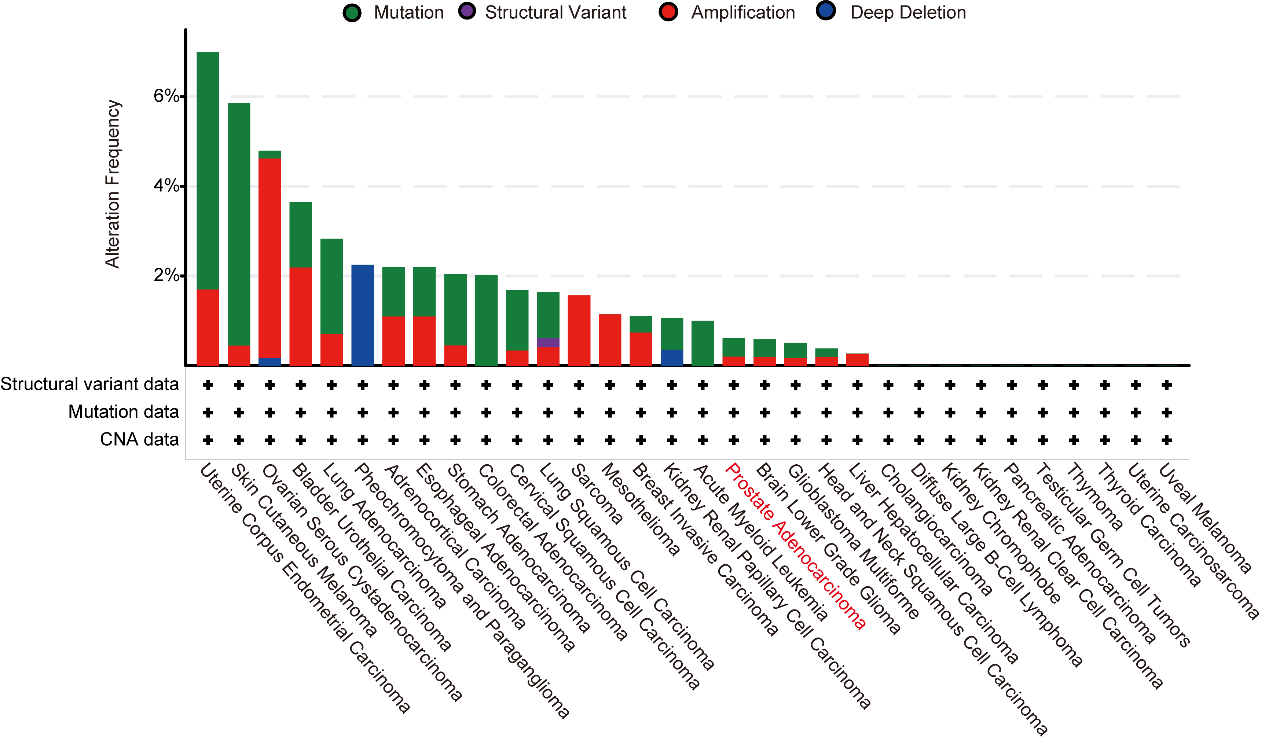


**Supplementary Figure 1**

Mutation feature of KIF2C in pan-cancer.


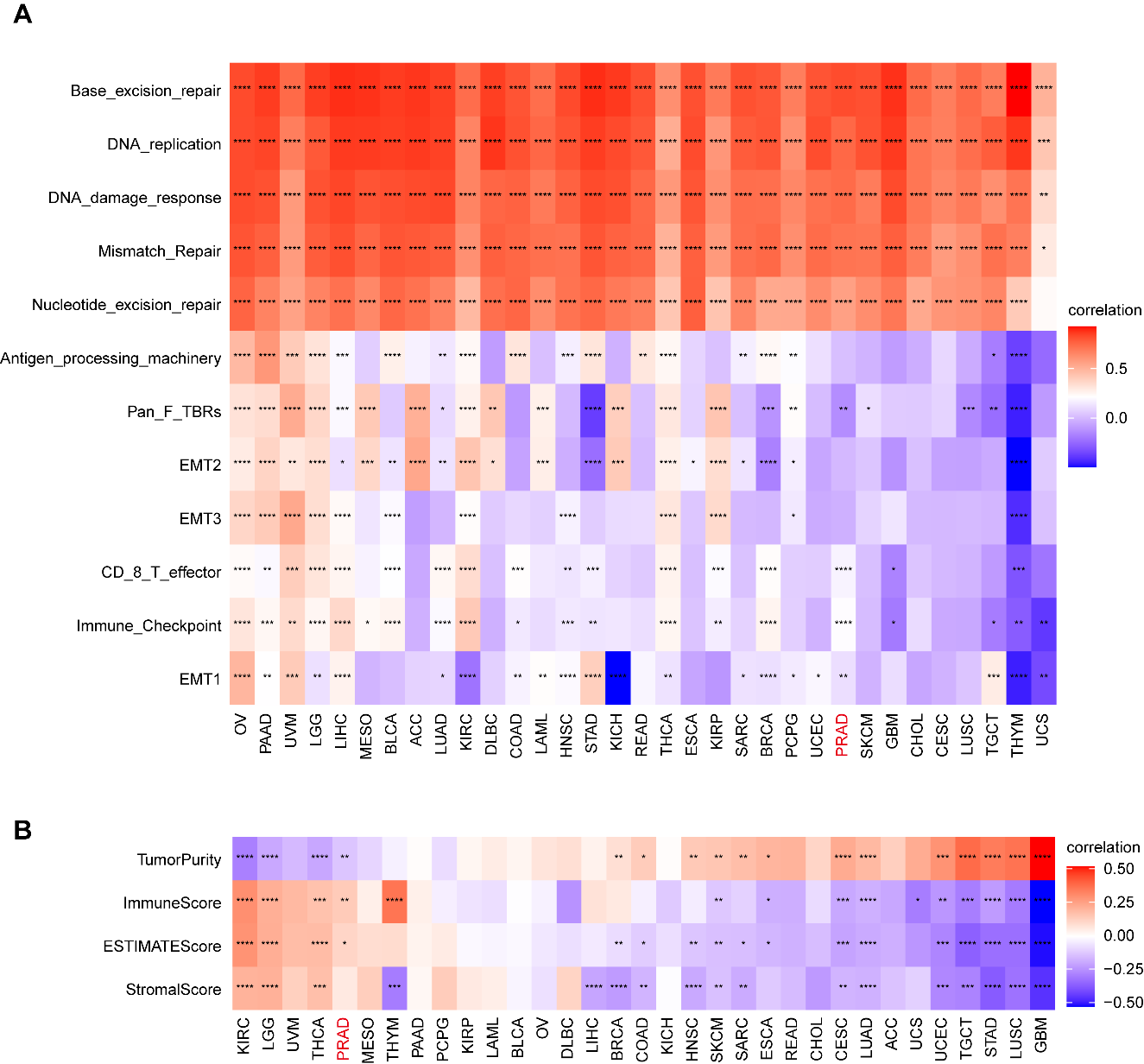


**Supplementary Figure 2**

Correlation of KIF2C expression with TME in pan-cancer. (A) The signaling pathway related to high KIF2C expression in pan-cancer. (B) Correlation between KIF2C and estimate scores, immune scores, and tumor purity in pan-cancer. **P* < 0.05, ***P* < 0.01, ****P* < 0.001, *****P* < 0.0001.


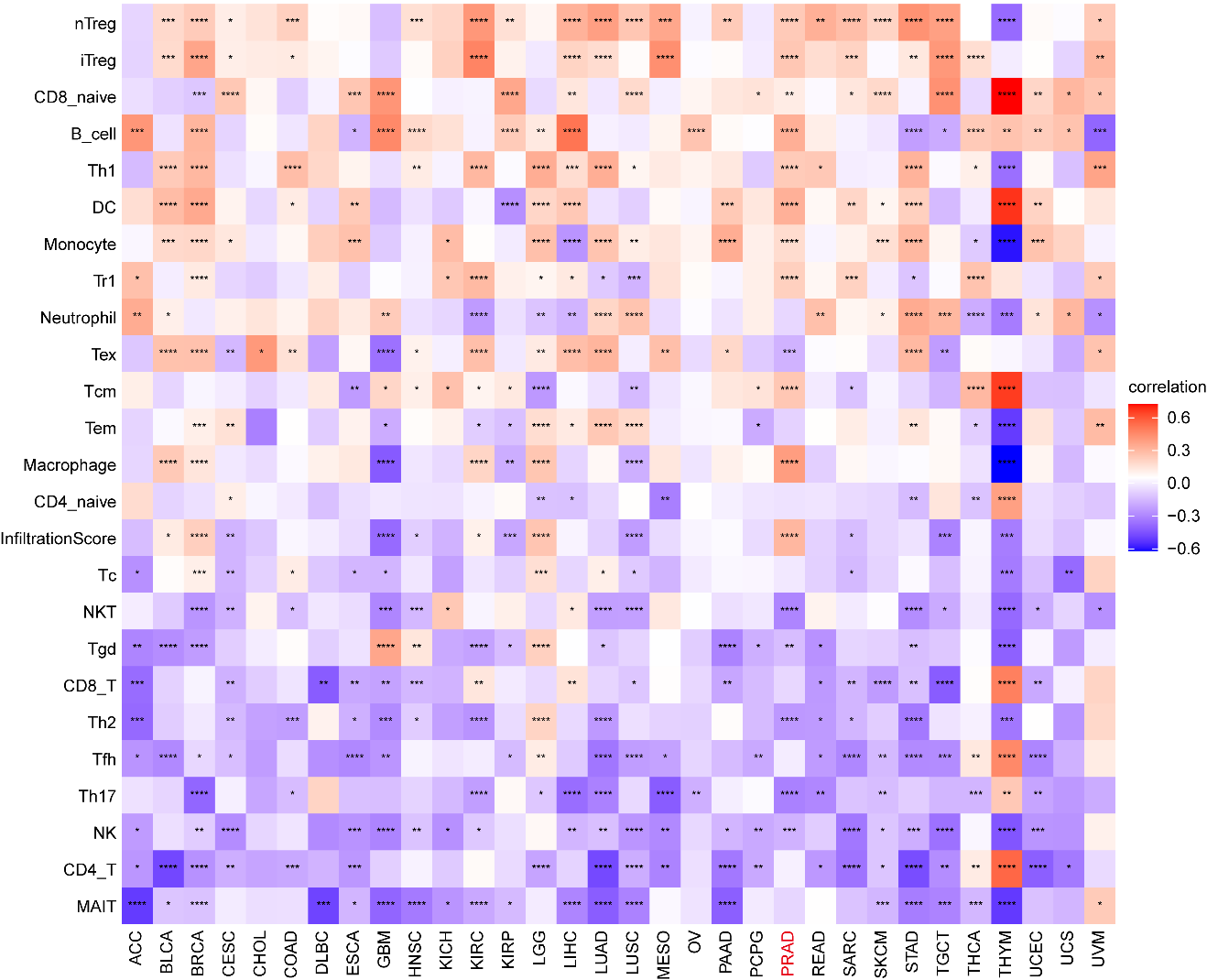


**Supplementary Figure 3**

KIF2C expression is correlated with tumor immune cell infiltration in pan-cancer. *P < 0.05, **P < 0.01, ****P* < 0.001, *****P* < 0.0001.
